# Demographic characteristics of those who cycle commute and the effect access to on- and off-road cycle paths has on cycle commute uptake.

**DOI:** 10.23889/ijpds.v9i5.2885

**Published:** 2024-09-18

**Authors:** Laurie Berrie, Chris Dibben, Zhiqiang Feng, Tom Clemens, Lee Williamson

**Affiliations:** 1 University of Edinburgh

## Objectives

Increasing cycle commute uptake could be an important approach to improving worker health and reducing carbon emissions, congestion and air pollution. In order to increase cycle commute uptake, we must understand the characteristics of those who already cycle to work and the aspects of physical infrastructure that could promote cycling.

## Approach

In this work, we use administrative data pertaining to those in employment aged 16-74 years living and working in Glasgow and Edinburgh, Scotland on the night of the 2011 population census. We calculate distances to the nearest cycle path for each individual from cycle route data from around the time of the census that were made computer readable. We looked at the characteristics of those who cycled to work and how proximity to nearest cycle path differed between groups along with how distance from place of residence and place of work to nearest cycle path influenced cycle commute uptake.

## Results

We show how the propensity to cycle commute differs across population characteristics such as sex, age and the National Statistics Socio-economic classification and how the interaction of these factors affects propensity to cycle commute. We also show the ways in which living and working closer to on- and off-road cycle paths increases the likelihood of cycle commuting.

## Discussion

The results of this study can be used to inform the most promising groups to aim cycling interventions at thereby improving health and environmental outcomes.

